# Dynamics of SARS-CoV-2 Immunoglobulin G Antibody Among Hospitalized Patients and Healthcare Workers During the Delta Wave in Bangladesh

**DOI:** 10.7759/cureus.82175

**Published:** 2025-04-13

**Authors:** Md. Abdul Matin, Chowdhury Adnan Sami, Md. Ashraful Hassan Anjan, Hasan M Rashed, Ashraful Hoque, Md Nazmul Hasan, Shohael Mahmud Arafat, Sunil K Biswas, Fazle R Chowdhury

**Affiliations:** 1 Department of Internal Medicine, Bangabandhu Sheikh Mujib Medical University, Dhaka, BGD; 2 Department of Blood Transfusion, Sheikh Hasina National Institute of Burn and Plastic Surgery, Dhaka, BGD

**Keywords:** bangladesh, community antibody, healthcare workers ab, hospitalized patients, sars-cov-2 igg antibody

## Abstract

Background: A reliable marker of COVID-19 past infection is the anti-spike IgG antibody (Ab) titer of the SARS-CoV-2 virus, which may be identified even one year from diagnosis. Serology can be effective in diagnosing asymptomatic infection and patients with negative reverse transcription polymerase chain reaction. Exploring these dynamics and the persistence of antibodies is vital to understanding the disease process.

Materials and methods: We designed this observational study to compare the seroconversion status of COVID-19 patients to healthy healthcare workers. The total number of participants was 272, including 58 hospitalized (recovered) patients, 153 healthcare workers (HCWs), and 61 control populations from the community. A total of 404 samples were taken for IgG titer measurements using the enzyme-linked immunosorbent assay method.

Results: Among 58 COVID-19 patients, 58.6% were men, and the male-to-female ratio was 1:1.41. All hospitalized patients had three months of persistence of Ab titer. The median IgG titer (interquartile range) optical density values for the hospitalized severe group on day 0, as well as during the fourth, eighth, and twelfth weeks, were 9.25 (6.01-11.893), 8.95 (5.52-12.42), 13.19 (12.26-13.26), and 12.99 (9.23-13.24), respectively. In comparison, the nonsevere group exhibited median values of 4.19 (3.08-9.89), 6.99 (4.32-9.75), 8.65 (5.83-13.08), and 12.48 (7.88-13.16), respectively. However, seroprevalence was higher in the control group (83.3%); in the HCWs, it was 54.9%. Although patients with severe disease had higher antispike IgG titers, no difference in titers was seen among age, gender, body mass index, or diabetes subgroups.

Conclusion: We observed Ab titer persistence up to three months in cases of COVID-19-recovered patients, whereas seroprevalence was high among HCWs and the control group during the Delta wave.

## Introduction

Studies have confirmed that IgM and IgG are neutralizing antibodies against SARS-CoV-2, as humoral responses play a vital role in eliminating it, and suggested the utilization of serologic testing for diagnosing COVID-19 [1,2]. IgM is the initial class of immunoglobulins and indicates early exposure to the antigen [3]. Patients may develop the anti-SARS-CoV-2 IgM antibody (Ab) four days after symptom onset. Before gradually decreasing, the Ab levels reach their highest point two to three weeks later [4,5]. Nevertheless, relying just on IgM positivity may not be a reliable diagnostic clue, as not all individuals produce detectable IgM antibodies [4], and they are only persistent for up to two months [3,6]. IgG is the most persistent Ab against the SARS-CoV-2 virus and is diagnosed, on average, 14 days after the symptoms first appear during the infection. Once detected, the Ab can remain stable for a period of 12 months [7-9].

Hence, serological tests identifying IgG antibodies offer a more dependable estimation of the prior COVID-19 infection. This is because these antibodies tend to persist longer after the viral infection has been cleared, making them a reliable indicator of past disease [10]. However, it is unclear how these responses would compare to those who are symptomatic or asymptomatic. Serological testing is, therefore, helpful for diagnosing suspected patients with negative reverse transcription polymerase chain reaction (RT-PCR) results and identifying asymptomatic infections [1].

Healthcare workers (HCWs) working at hospitals, who are at the frontlines of treating COVID-19, had a higher risk of infection [11]. By nature, they have a higher level of exposure to the virus compared to the general population, which puts them at a heightened risk of infection [12].

Identifying and estimating the seroprevalence among asymptomatic individuals and frontline HCWs and the frequency and timing of seroconversion in hospitalized COVID-19 patients by humoral Ab (IgG titer) response are essential for understanding disease dynamics. Therefore, in this study, we examined the frequency and timing of seroconversion among hospitalized (recovered) symptomatic COVID-19 patients and the SARS-CoV-2 seroprevalence among HCWs working at the COVID unit of Bangabandhu Sheikh Mujib Medical University (BSMMU). In this study, these factors were explored in the COVID unit of BSMMU.

## Materials and methods

Participant recruitment

We conducted this prospective observational study in BSMMU, Shahbag, Dhaka, from October 2020 to September 2021. Participants who enrolled in this study were categorized into three groups: Group A: patients aged ≥18 years with RT-PCR-positive COVID-19 and are within 22-42 days following the onset of symptoms, who required hospital admission; Group B: healthcare workers who served in the BSMMU COVID-19 unit for at least seven days of roster duty and fell between 22 and 42 days after service. HCWs were further subgrouped into doctors, nurses, and supporting staff groups; and Group C: a small asymptomatic population was taken for the control group.

Patients who were hospitalized and have recovered were tracked by telephone for subsequent follow-up. HCWs who were already COVID-19-positive were excluded from the study. Sociodemographic, epidemiological, clinical characteristics, and laboratory data were collected through a case record form.

We excluded those who received any type of COVID-19 vaccine, including even the first dose; pregnant or lactating mothers; individuals using current medication known to alter immunity, such as steroids, anticancer drugs, or biologics; and those who received plasma therapy or experienced concomitant critical illnesses, such as sepsis, acute kidney injury, or active malignancy, across all groups. Additionally, participants who were currently suffering from COVID-19-like illnesses or were known to be RT-PCR-positive for COVID-19 were also excluded from Groups B and C.

The primary aim was to assess IgG dynamics in hospitalized patients, while secondary goals include comparing seroprevalence among HCWs and the control group. We used an IgG Ab kit with enzyme-linked immunosorbent assay (ELISA)-based technology to measure the IgG titer (optical density, OD, value). For seroprevalence status, we have done the titer for Group B (HCW) and the control at a single time point. For Group A, to explore seroconversion, a series of tests was done at day 0 (between 22 and 42 days after symptom onset), then at the fourth, eighth, and twelfth weeks after day 0.

Laboratory technique

After obtaining informed written consent, 3 mL of blood was collected from the subjects. The samples were rendered inactive by being subjected to a temperature of 56°C for 30 minutes. After separating the plasma using centrifugation, the samples were stored at -20℃ until further testing. All samples underwent only one freeze-thaw cycle to minimize degradation of Ab proteins.

To investigate the humoral immune response, we measured the levels of total IgG antibodies that specifically recognize the spike protein of the virus in the plasma. This was done using an ex vivo ELISA technique. The test was done at the Sheikh Hasina National Institute of Burn and Plastic Surgery (SHNIBPS) with the Kewei COVID-19 IgG Ab ELISA Test Kit (Beijing Kewei Clinical Diagnostic Reagent Inc., China); clinical sensitivity and specificity of the kit are 90.83% and 99.2%. The samples were transferred to SHNIBPS following standard sample transfer procedures.

Ethical considerations and data management

The Institutional Review Board of BSMMU (registration number BSMMU/2021/159) gave ethical approval. The entirety of the research data was encoded and maintained in a secret manner. Every patient was assigned a distinct identification number to ensure the protection of their confidentiality and anonymity. The principal investigator was responsible for maintaining the confidentiality of all patient information, including laboratory test results.

Statistical analysis

Data analysis was carried out using the Statistical Package for Social Sciences version 25 for Windows (SPSS Inc., Chicago, IL) and GraphPad Prism version 8 (Dotmatics, Boston, MA). Qualitative variables (sex, comorbidities, etc.) of this study were expressed as a percentage. Quantitative variable (e.g., age) expressed as mean ± standard deviation (SD)/median with interquartile range. Qualitative data were analyzed using chi-square or Fisher's exact test where appropriate, and quantitative data were obtained using Student's t-tests or Mann-Whitney U tests. For all statistical tests, a p value of <0.05 was considered statistically significant.

## Results

Enrolment status of the participants

We have enrolled 65 recently recovered COVID-19 patients since the mass vaccination program started in Bangladesh. Among them, seven patients were excluded (one died and six patients denied subsequent follow-up). Therefore, finally, 58 hospitalized (recovered) patients were followed up at four time points, but the first point follow-up consisted of 56 patients, as two patients missed the first point follow-up. For Group B, 153 HCWs were enrolled; among them, 50 were doctors, 52 were nurses, and 51 were support staff. A total of 61 asymptomatic participants (Group C) were enrolled from the surrounding community as controls. For Groups B and C, samples were collected at a single time point. In Group A, some of the patients missed the subsequent follow-up at different time points due to the ongoing lockdown and vaccination program. Patients were further divided into nonsevere groups consisting of mild and moderate disease of COVID-19 and severe groups consisting of severe and critical COVID-19.

**Figure 1:**
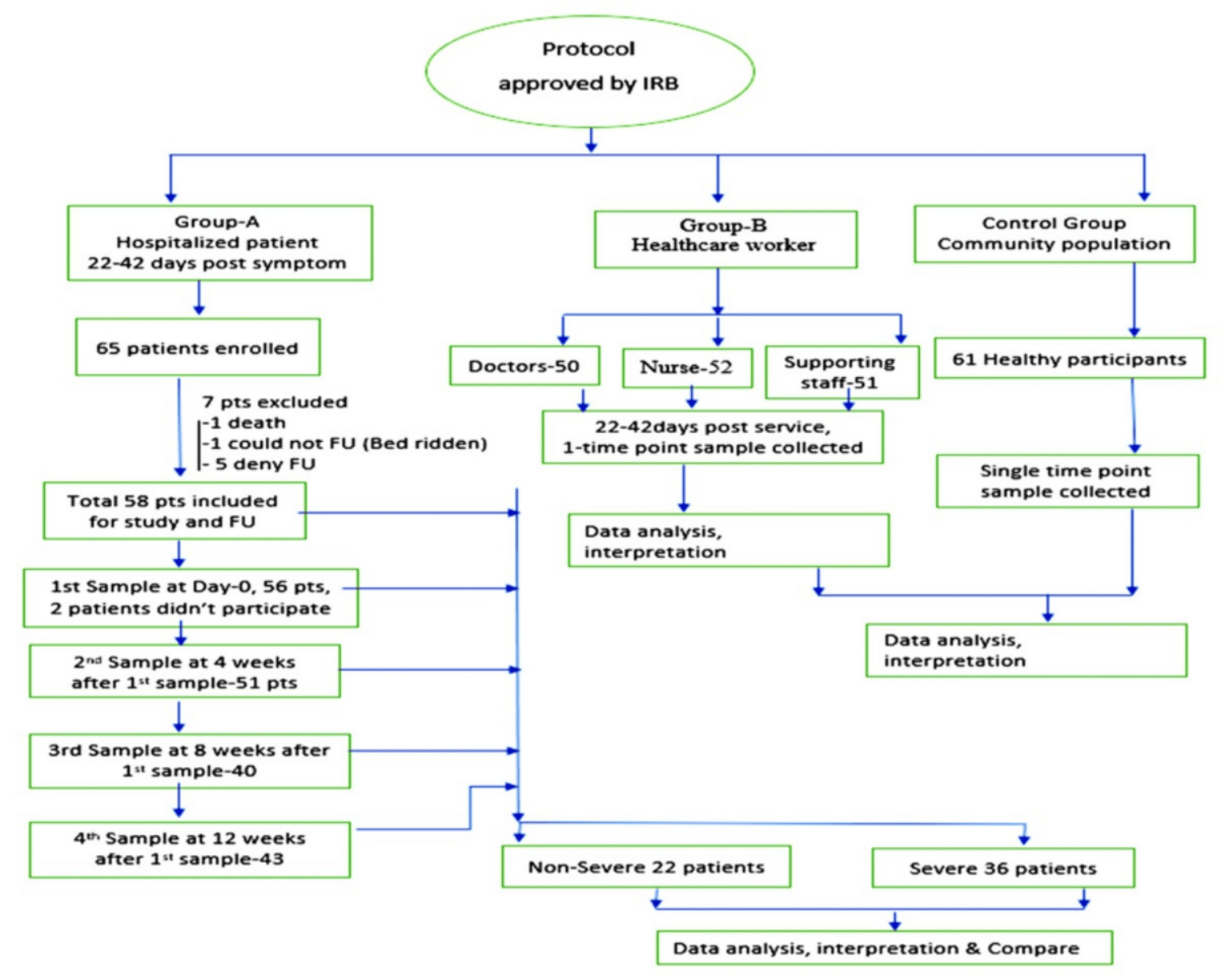
Consort diagram representing the flow of enrollment and sample collection IRB: institutional review board; FU: follow-up

Of the 272 participants, 404 samples were taken for IgG titer measurement. Among the recovered patients, on day 0 (22-42 days), 56 patients, at four weeks, 51 patients, at eight weeks, 40 patients, and at 12 weeks, 43 patients gave their blood samples (Figure 1).

Clinical profile and demographics of the participants

The most common symptoms at hospital admission were fever 54 (93.1%), fatigue 55 (94.8%), cough 50 (86.2%), dyspnea 36 (62.1%), body ache/myalgia 38 (65.5%), and sore throat 22 (37%). All symptoms were equivalent in both groups except dyspnea, which was more frequent in the severe group (p = 0.002) (Table 1).

**Table 1:** Baseline sociodemographic characteristics of the hospitalized (recovered) patients ^a^Chi-square test was done ^b^Fisher's exact test was done

Characteristics	All patients (n = 58)	Nonsevere (n = 22)	Severe (n = 36)	Chi-square value/Fisher's exact value	p value
Clinical symptoms on admission					
Fatigue	55 (94.8%)	20 (90.9%)	35 (97.2%)	1.110	0.551^b^
Fever	54 (93.1%)	21 (95.5%)	33 (91.7%)	0.305	1.00^b^
Anorexia	52 (89.7%)	18 (81.8%)	34 (94.4%)	2.347	0.187^b^
Cough	50 (86.2%)	18 (81.8%)	32 (88.9%)	0.574	0.462^b^
Nausea	44 (75.9%)	16 (72.7%)	28 (77.8%)	0.190	0.663^a^
Taste loss	41 (70.7%)	14 (63.6%)	27 (75%)	0.851	0.356^a^
Body ache/myalgia	38 (65.5%)	17 (77.3%)	21 (58.3%)	2.168	0.141^a^
Smell loss/anosmia	37 (63.8%)	14 (63.6%)	23 (63.9%)	0.000	0.985^a^
Dyspnea	36 (62.1%)	8 (36.4%)	28 (77.8%)	9.948	0.002^a^
Headache	31 (53.4%)	15 (68.2%)	16 (44.4%)	3.092	0.079^a^
Chest pain	23 (39.7%)	7 (31.8%)	16 (44.4%)	0.910	0.340^a^
Palpitation	23 (39.7%)	7 (31.8%)	16 (44.4%)	0.910	0.340^a^
Diarrhea	22 (37.9%)	6 (27.3%)	16 (44.4%)	1.710	0.191^a^
Sore throat	22 (37.9%)	11 (50%)	11 (30.6%)	2.193	0.139^a^
Nasal congestion	18 (31%)	10 (45.5%)	8 (22.2%)	3.444	0.063^a^
Abdominal pain	17 (29.3%)	5 (22.7%)	12 (33.3%)	0.741	0.389^a^
Vomiting	15 (25.9%)	4 (18.2%)	11 (30.6%)	1.090	0.296^a^
Others (hematuria, oliguria)	2 (3.4%)	0	2 (5.6%)	0.622	-
Management					
Oxygen	36 (62%)	0	36 (100.0%)	53.87	-
Antiviral	31 (53.4%)	3 (13.6%)	28 (77.8%)	22.576	0.0001^a^
Antibiotic	54 (93.1%)	18 (81.8%)	36 (100%)	7.037	0.017^b^
Anticoagulant	46 (79.3%)	10 (45.5%)	36 (100%)	24.759	0.0001^b^
Steroid	44 (75.9%)	8 (36.4%)	36 (100.0%)	30.198	0.0001^a^

Severe patients received treatment with oxygen 36 (100%), antibiotics 36 (100%), anticoagulants 36 (100%), steroids 36 (100%), antivirals 28 (77.8%), and tocilizumab 3 (8.3%). Antiviral, steroid, and anticoagulant use was significantly higher in severe cases (p < 0.05) (Table 1).

The median age of recovered hospitalized patients was 45.5 (33-57) years, and 34 (58.6%) of the patients were men, while their body mass index (BMI) was 25.4 (23.39-27.42). Among HCWs, male patients were 84 (54.9%), while their age was 30 (25-33) and BMI was 24 (21.53-25.71). In the control population, 50 (82%) were men, while their median age was 27 (25-32) and BMI was 23.8 (22.6-24.97).

In the case of occupation, among the hospitalized recovered patients, 31 (53.4%) were service holders, 10 (17.2%) were businesspeople, 13 (22.4%) were homemakers, and 4 (6.9%) were students. Among the control group, 12 patients (19.6%) were in service, 10 patients (16.4%) were businesspeople, four patients (6.6%) were homemakers, 16 patients (26.2%) were students, and 19 (31.1%) were workers. Among the workers, eight patients (42.1%) were barbers, six patients (31.5%) were pharmacy workers, and five patients (26.31%) were food delivery workers (Table 2).

**Table 2:** Baseline clinical characteristics and management data of the COVID-19-recovered patients IQR: interquartile range; BMI: body mass index

Characteristics	All (n = 272)	Recovered patients (n = 58)	Healthcare workers (n = 153)	Control (n = 61)
Age (years), median (IQR)	31 (26-36)	45 (33-57)	30 (25-33)	27 (25-32)
Sex				
Male	168 (61.8%)	34 (58.6%)	84 (54.9%)	50 (82%)
Female	104 (38.2%)	24 (41.4%)	69 (45.1%)	11 (18%)
BMI, median (IQR)	24.2 (22.06-25.98)	25.4 (23.39-27.42)	24 (21.53-25.71)	23.8 (22.6-24.97)
Occupation				
Service	192 (70.6%)	31 (53.4%)	153 (100%)	12 (19.6%)
Business	20 (7.4%)	10 (17.2%)	0	10 (16.4%)
Homemaker	13 (4.8%)	13 (22.4%)	0	4 (6.6%)
Student	19 (7%)	4 (6.9%)	0	16 (26.2%)
Workers	19 (7%)	0	0	19 (31.1%)

Baseline investigation

The severe patient had a significantly higher erythrocyte sedimentation rate (ESR), high C-reactive protein (CRP), high leukocyte counts, high neutrophil count, low lymphocyte counts, high blood sugar, elevated neutrophil-lymphocyte ratio (NLR), high ferritin, high alanine transaminase, and high computed tomography (CT) score than the nonsevere group. However, between the groups, there was no significant change in D-dimer (p = 0.327) and lactate dehydrogenase levels (p = 0.241) (Table 3).

**Table 3:** Baseline investigation profile of COVID-19-recovered patients An independent sample t-test was done HB: hemoglobin; ESR: erythrocyte sedimentation rate; WBC: white blood cell; NLR: neutrophil-lymphocyte ratio; CRP: C-reactive protein; RBS: random blood sugar; ALT: alanine transaminase; LDH: lactate dehydrogenase; HRCT: high-resolution computed tomography

Investigation	All patients (n = 58), mean ± SD	Nonsevere (n = 22), mean ± SD	Severe (n = 36), mean ± SD	t-test value	p value
HB	12.22 ± 1.45	12.62 ± 0.77	11.98 ± 1.71	1.925	0.060
ESR	39.76 ± 22.42	31.68 ± 14.81	44.69 ± 24.92	-2.494	0.016
WBC	8,641.6 ± 3,713.9	7,417.3 ± 1,855.1	9,389.7 ± 4,345.2	-3.626	0.021
Neutrophil	74.03 ± 9.67	68.68 ± 7.81	77.31 ± 9.32	-3.786	0.001
Lymphocyte	19.69 ± 8.41	24.14 ± 6.79	16.97 ± 8.22	3.594	0.001
NLR	5.47 ± 6.45	3.92 ± 1.85	6.79 ± 7.84	-2.573	0.044
Platelet	252,336 ± 128,209	179,204 ± 44,542	297,027 ± 141,997	-4.620	0.0001
CRP	36.62 ± 41.22	13.34 ± 15.92	50.84 ± 45.45	-4.518	0.0001
Ferritin	531.5 ± 533.58	183.95 ± 100.4	743.81 ± 579	-5.663	0.0001
D-dimer	0.6 ± 1.03	0.43 ± 0.21	0.70 ± 1.29	-0.990	0.327
RBS	9.6 ± 4.3	8.1 ± 3.5	10.56 ± 4.48	-2.193	0.03
Serum creatinine	1.07 ± 0.53	0.94 ± 0.17	1.15 ± 0.65	-0.459	0.146
ALT	55.59 ± 39.05	39.14 ± 19.67	65.64 ± 44.47	-3.327	0.003
LDH	414.8 ± 198.5	291.0 ± 129.7	445.7 ± 204.7	-1.229	0.241
Procalcitonin	0.148 ± 0.21	0.05 ± 0	0.16 ± 0.22	-0.878	0.39
HRCT score	11.2 ± 5.4	7 ± 3.3	12.23 ± 5.5	-3.327	0.003

IgG titer in hospitalized patients

We tested 56 hospitalized and subsequently recovered patients for humoral immune response at the first point by measuring antispike IgG Ab titer at four time points. Out of 56 patients, 54 developed measurable IgG titers; one patient developed an IgG titer at four weeks, and another developed it at eight weeks. The interpretation of the OD value was <1.1 for negative and ≥1.1 for positive. The lowest titer was 0.5 at day 0, and the highest Ab titer was 13.6 at eight weeks.

**Figure 2:**
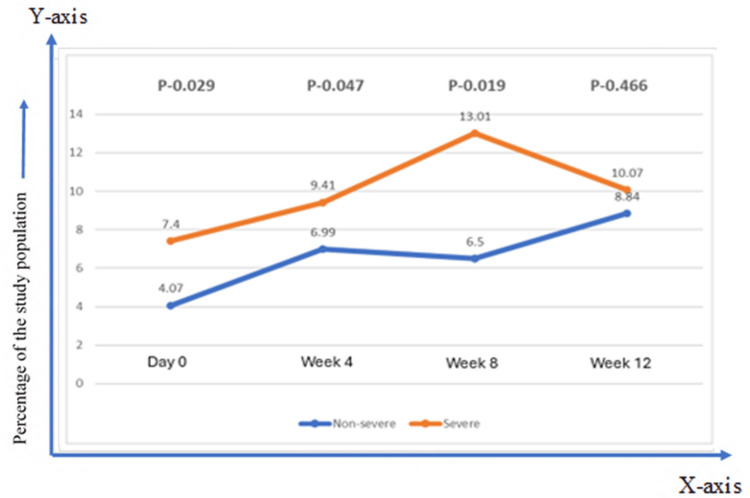
Comparison of immune response and dynamics of antispike antibody (median titer) against SARS CoV-2 in the severe and the nonsevere group The Mann-Whitney U test was applied

In the nonsevere group, IgG titer gradually increased to 12 weeks, while in the severe group, IgG titer gradually increased to eight weeks and then slightly declined at 12 weeks. Antispike IgG Ab titer was higher at all time points in the severe, with a significant p value in all the samples (Figure 2).

IgG titer among HCWs and the control population

**Figure 3:**
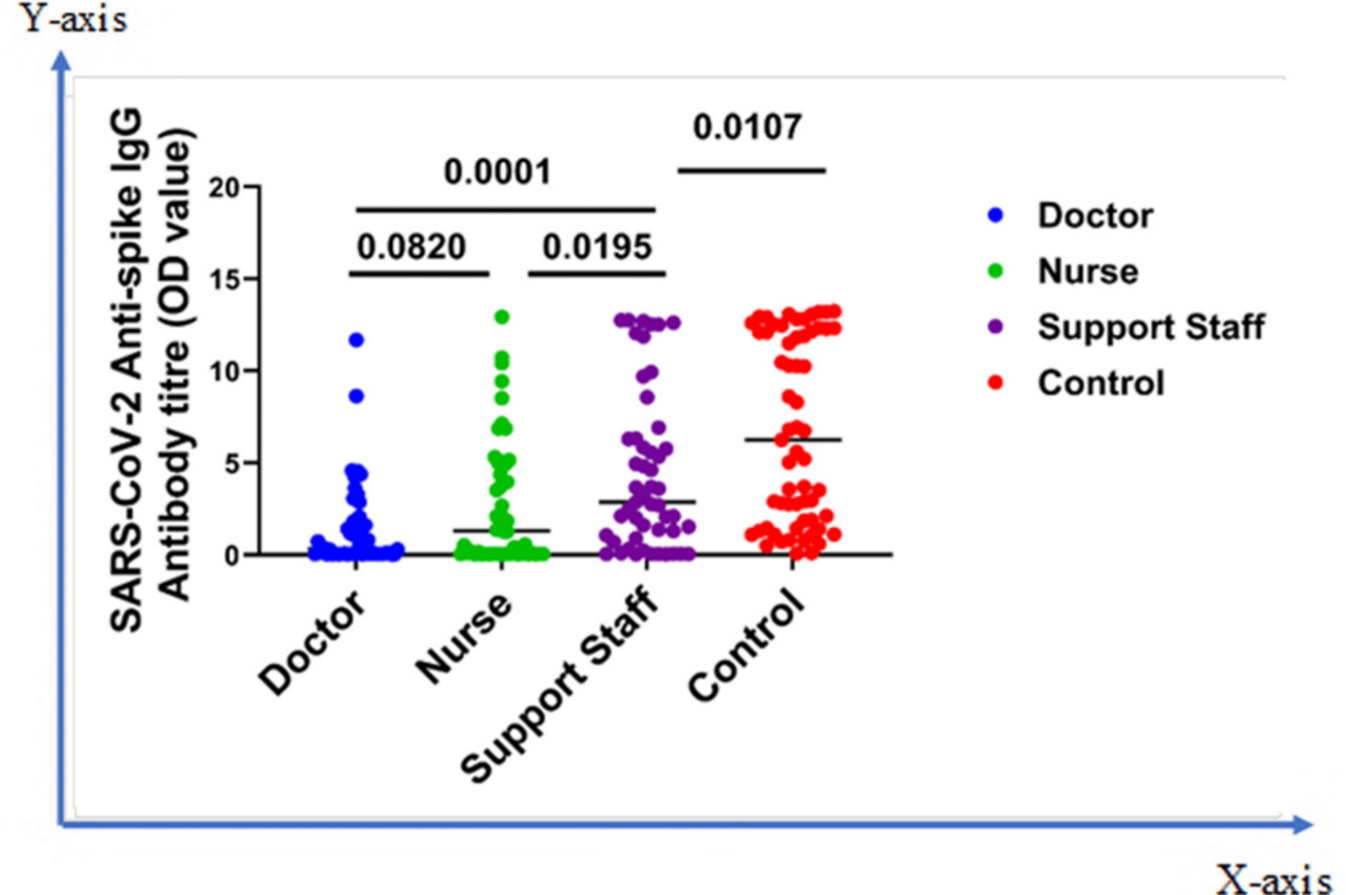
Comparison of immune response among different categories of asymptomatic HCWs (doctor, nurse, and support staff) and control The Mann-Whitney U test was applied HCWs: healthcare workers; OD: optical density

Figure 3 shows the IgG Ab response status among different categories of asymptomatic HCWs who recently finished a one-week COVID-19 hospital duty rotation. The overall seropositivity was 38% for doctors, 53.8% for nurses, and 72.5% for support staff. The mean Ab titers of the doctors, nurses, and support staff were 1.47 ± 0.33, 2.71 ± 0.46, and 4.46 ± 0.60, respectively. A significant difference was revealed between doctors and support staff (p = 0.0001), and nurses and support staff (p = 0.0195). No difference was seen between doctors and nurses (p = 0.082). A significant difference (p = 0.0107) was also seen between support staff and control population, where a higher Ab level was observed among the control population (Figure 3).

IgG titer difference among different subgroups of the population

**Figure 4:**
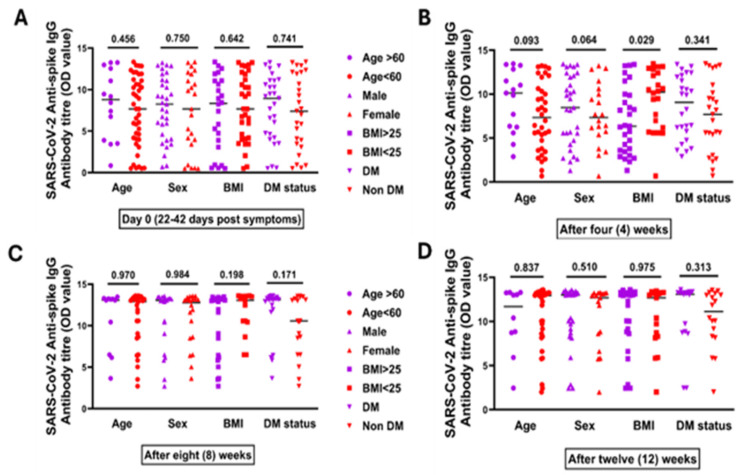
Antispike IgG antibody titer among recovered COVID-19 patients according to age, sex, BMI, and DM status at different time points. (A) Day 0 (which is 22-42 days after first COVID-19 symptoms), which showed the median antispike IgG antibody titer, in terms of age (≥60 vs. <60 years), BMI (≥25 vs. <25 kg/m2), sex (male vs. female), and diabetes status (DM vs. non-DM). (B) After four weeks, which showed median antispike IgG antibody titer in terms of age, BMI, sex, and diabetes status. (C) After eight weeks, which showed median antispike IgG antibody titer in terms of age, BMI, sex, and diabetes status. (D) After 12 weeks, which showed median antispike IgG antibody titer in terms of age, BMI, sex, and diabetes status The Mann-Whitney U test was done DM: diabetes mellitus; IgG: immunoglobulin G; OD: optical density; BMI: body mass index

Figure 4 illustrates the median antispike IgG antibody titers measured at four time points: day 0 (corresponding to 22-42 days after symptom onset), and subsequently at four, eight, and twelve weeks. Titer differences were shown in terms of age (≥60 vs. <60 years), BMI, sex (male vs. female), and diabetes status (diabetes mellitus, DM, vs. non-DM). No significant disparity was seen between severe vs. nonsevere at any time point except for BMI after four weeks, which signifies that obese patients tend to mount a higher Ab response compared to nonobese (Figure 4).

## Discussion

We conducted this study to assess the immune response and changes in levels of SARS-CoV-2-specific IgG antibodies in patients who were hospitalized and later recovered, HCWs, and healthy individuals in the nearby community. In this study, among the hospitalized patients, there were more men than women, which was similar to studies done in China [4,13,14] but dissimilar to others where the female participants were more [2,8,10,15]. This is probably related to the limited access to healthcare for women in low- and middle-income countries like Bangladesh.

The clinical manifestations of both groups were similar in other studies [10,13,16-18]. Notably, the neurological symptoms, abdominal symptoms, and cardiac symptoms were higher in these patients than in the previous studies. The common symptoms of the Delta variant were headache, cold, sore throat, anosmia, abdominal pain, chest pain, and palpitations [19,20]. The study was done from March to August 2021, when the Delta variant infection was at its peak in Bangladesh [21], which explains the predominance of the symptoms in this study.

We found that the severe patient group had a higher mean (SD) ESR, CRP, leukocyte counts, neutrophil count, NLR, blood sugar, ferritin, serum glutamic pyruvic transaminase, CT score, and low mean lymphocyte counts than the nonsevere group, which was matched with other studies [13,18,19,22].

Both severe and nonsevere cases mounted considerable amounts of antispike IgG Ab titer, which increased over time and peaked around 8-12 weeks. Severe patients developed antibodies earlier than nonsevere patients. Individuals with more severe disease tended to have larger peak Ab responses or positive associations at one and two months after the onset of symptoms, like those seen in much previous research [8,9,15,23]. Some studies reported that anti-SARS-CoV-2 IgG antibodies persisted for three months and then declined [14,15,24,25]. Although there was a slight decline in Ab titer for severe patients, the level was still considerably high in this study. Few studies have reported the sustainability of Ab titer for up to six to seven months [26,27]. Asymptomatic patients maintained Ab titers for up to five to six months, whereas symptomatic patients maintained Ab titers for up to 12 months [9]. Some studies also showed that IgG titers in the symptomatic patients were considerably greater than in the asymptomatic patients at all sampling periods [17,28,29]. Although this study did not measure the Ab titer beyond three months, the high value presumably signifies its persistence for a similar duration, like others.

The study found that the seroprevalence among HCWs was 54.9%, with supporting staff at 72.54%, nurses at 53.84%, and doctors at 38%. This rate is markedly greater than the rates seen in similar studies performed in other locations. Iversen et al. found a significantly higher seroprevalence among HCWs in the dedicated COVID-19 ward, at 7.19%, compared to other nonfrontline in-hospital HCWs, which was 35% [11]. Reports from Asian countries showed that HCWs constituted over 20% of presumptive occupation-related cases [30]. The prevalence of asymptomatic HCWs in a tertiary-care hospital in India was 11.9% [16], 13.2% in Pakistan [31], 13.7% in the United States [32], and 17.14% in the United Kingdom [33]. However, this study found the prevalence to be much higher. We assumed that this high seroprevalence of HCWs in Bangladesh might be due to breaches of infection prevention control (IPC) measures, reluctance to use personal protective equipment (PPE), and lack of proper training about IPC among support staff, nurses, cleaners, ward boys, and others.

Among healthcare professionals, the incidence was slightly greater among nurses (3.4%) and wardens (3.4%) compared to physicians (2.6%) in the Spanish study [34]. A study conducted in China also discovered a greater seroprevalence among nurses than physicians [35]. In a separate study carried out in Los Angeles, it was found that infections among nurses constituted over 50% of all COVID-19 cases among HCWs [36]. This trend of seropositivity in different HCW categories was consistent with our study. This is probably explained by the lack of training on IPC and PPE usage among the support staff in the BSMMU COVID unit.

In this study, humoral immune response at a single time point was also compared between high-risk groups (HCWs) and surrounding populations. It has been found that asymptomatic patients were associated with lower virus-specific IgG levels and a higher seropositivity rate in the early convalescent phase [1]. Similarity has been observed in this study. In between the asymptomatic control group (surrounding population) and the HCWs who worked in the COVID-19-dedicated hospital, there was a significant difference in the IgG Ab titers. This means that in Bangladesh, lots of people were asymptomatic during the Delta wave. In other studies, the overall seroprevalence of the general population was found to be 36%, with the highest positivity in industrial employees: 50.5% in Pakistan [31], 22% for Iran [37], and 22%-33% for India [38], which was inconsistent with our study. A previous study in Bangladesh showed that the seroprevalence of COVID-19 among slum dwellers in Dhaka is 68% [39], which is close to our study findings.

The fact that HCWs had a lower Ab positivity rate than the control group may sound somewhat counterintuitive; there could be several reasons. First, infection control measures in healthcare settings would have been implemented more stringently, thereby reducing exposure risk among HCWs compared to the general community, where transmission was likely more widespread. In addition, HCWs may have also had previous mild or asymptomatic infections and thus lower Ab titers that fell below the detection threshold. In addition, HCWs were most likely to be infected earlier in the pandemic; their Ab levels might have waned more substantially by the time of sampling.

We also checked the IgG Ab titer value according to age, sex, diabetes status, and BMI. There was no notable disparity in the duration of IgG Ab levels seen among different age groups (above 60 years and below 60 years), genders (male and female), individuals with diabetes compared to those without diabetes, and individuals with a BMI over 25 and below 25. These results were consistent with previous studies that found no notable disparities in SARS-CoV-2 antispike IgG Ab levels between male and female individuals recovering from the illness as time progressed [7,17,24]. Simonnet et al. found a higher BMI associated with severe disease [25]. Based on this relationship, a similar higher antispike IgG Ab titer was seen in patients with a higher BMI in another study [8]. In a study done at this study site (BSMMU), Ali et al. observed higher antispike IgG (including neutralization titer) among lean patients [39]. After four weeks, we also observed significantly higher IgG Ab responses in lean patients; however, differences were insignificant over time (8 and 12 weeks). In obesity, viral clearance can be slow due to multiple reasons, such as dysregulated cytokine response, chronic inflammation, altered T-cell homeostasis, and impaired cellular immunity [39]. The same study revealed higher antispike IgG in diabetes patients without any significant difference in neutralization capacity [39]. In this study, we have also noticed a higher trend among diabetes patients without any significant difference over time.

This study has multiple drawbacks. First, it was a single-center study with a small sample size, which might limit its generalizability and thorough examination of the Ab responses and seroprevalence status. We made every effort to minimize preanalytical variability, but we did not formally evaluate interassay variability across testing batches, which can cause minor measurement variation. We followed up with the patient for three months; however, it would have been better to follow up for more months to have a clear idea of the persistence of antibodies in the recovered patients. Meanwhile, a mass vaccination program started throughout the country; therefore, the dropout rate was high. Although the Delta variant was dominant during that time, we did not confirm the variant with which our cohort was infected. The absence of variant information limits the ability to compare the findings with other studies conducted during different periods or in different locations. However, the Delta variant was later superseded by the Omicron variant; our study provides a strong baseline for important kinetics of natural infection-induced immunity. Finally, the control group's age and BMI did not match those of the other groups.

## Conclusions

In this study, we showed the effects of humoral immune response during the Delta era among hospitalized recovered COVID-19 patients, frontline HCWs, and asymptomatic patients in Bangladesh. Our results showed that hospitalized patients mount a robust antispike IgG Ab response that lasts at least four months. In fact, our analysis excluded immunized hosts so that the immune responses we observed were purely due to infections. Afterward, Ab titers in the severe and the nonsevere disease groups peaked at about eight weeks but then had a downward trend. Among health care workers, support staff had the highest Ab levels, possibly due to variations in infection control protocols. Furthermore, the control group had much higher seroprevalence during the study period, indicating extensive community transmission during the Delta wave. In the IgG levels, there was no difference between different age groups, gender, BMI, or diabetes status. Although subsequent variants and vaccination are likely to impact immune responses, our data provide insight into baseline immune kinetics. Future comparative studies of the infection-induced immunity among different variants and vaccine-induced immune responses will provide a comprehensive understanding of the Ab kinetics during the postvaccination era.
